# Water in nigella oil microemulsion for enhanced oral bioavailability of linagliptin

**DOI:** 10.1007/s13346-024-01613-x

**Published:** 2024-05-13

**Authors:** Rania K. Eid, Mona F. Arafa, Gamal M. El Maghraby

**Affiliations:** 1 Department of Pharmaceutical Technology, Faculty of Pharmacy, Tanta University, Tanta, Egypt; 2 Department of pharmaceutics, Faculty of pharmacy, University of Tabuk, Tabuk, Saudi Arabia

**Keywords:** Linagliptin, Nigella oil, Microemulsion, Oral absorption, Class III drugs, Hypoglycemic effect

## Abstract

Linagliptin is hydrophilic antidiabetic with poor oral bioavailability due to poor permeability and pre-systemic metabolism. The objective was to assess w/o microemulsion for enhanced oral bioavailability of linagliptin. Nigella oil was used as oily phase based on its reported antidiabetic effect. Isopropyl myristate (IPM) or capryol were combined with nigella oil to impart intestinal membrane permeabilizing abilities. Pseudoternary phase diagrams were constructed utilizing nigella oil in presence and absence of isopropyl myristate or capryol as oily phase using Tween 60 as surfactant. W/O microemulsion formulations were selected from the constructed phase diagrams and linagliptin was loaded in the internal aqueous phase at a concentration of 0.5 mg/ml. The prepared formulations were physically evaluated and linagliptin in vitro release was monitored. Eventually, the in vivo hypoglycemic effect was assessed using diabetic rats. The developed microemulsions were of w/o type and exhibited Newtonian flow behavior with nigella/capryol microemulsion recording the lowest viscosity. The recorded droplet size values were 104.9, 121.2 and 86.4 nm for nigella, nigella/IPM and nigella/capryol microemulsions, respectively. All microemulsion formulations showed slower drug release rate compared with aqueous suspension with nigella/capryol microemulsion showing the highest release rate compared to other microemulsions. Release data from microemulsion best fitted to Higuchi model. In vivo oral hypoglycemic activity measurement reflected a more intensified hypoglycemic effect with rapid onset after oral ingestion of microemulsion compared to linagliptin dispersion. Nigella oil/IPM-based microemulsion was ranked as the most effective. The investigation highlighted the feasibility of w/o microemulsion for enhanced oral bioavailability of hydrophilic drugs like linagliptin.

**Graphical abstract:**

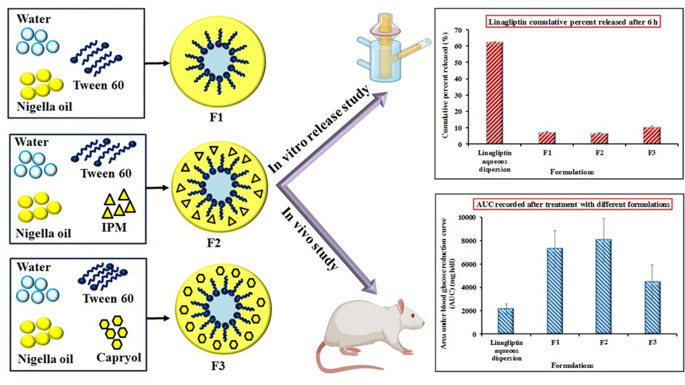

## Introduction

Linagliptin is potent dipeptidyl-peptidase 4 inhibitors. It is one of much effective dipeptidyl-peptidase 4 inhibitors that is employed either as mono or combined therapy for treating type II diabetes mellitus [1–3]. However, linagliptin has low oral bioavailability recording only 29.5% which attributed to its first-pass metabolism, poor permeation, and intestinal P-glycoprotein (P-gp) efflux [3–7]. Accordingly, the improvement of oral bioavailability of linagliptin has been investigated by researchers with various strategies being adopted. The efficacy of P-gp inhibitors has been exploited by the researchers as an approach for enhancing oral bioavailability of linagliptin. The studies indicated that natural compounds like gallic acid and ellagic acid were able to augment the bioavailability of linagliptin after oral administration and the reason was linked with P-gp inhibitory effect [7]. In addition, other investigators demonstrated that co-perfusion of linagliptin with P-gp inhibitors enhanced the intestinal permeation of linagliptin. The employed inhibitors were either drugs like carvedilol and atorvastatin or excipients like bile salts [8]. Recently, nanotechnology-based systems have been also investigated as a possible strategy for improving linagliptin oral bioavailability, but limited number of studies are available in literature. Dispersion of solid lipid nanoparticles loaded with linagliptin was in vivo evaluated and demonstrated enhancement of oral bioavailability compared to pure drug [9]. Nishu et al. incorporated linagliptin in niosomes and studied linagliptin permeability using chicken intestinal sac [10].

Linagliptin belongs to BCS class III drugs imparting high solubility and poor permeability. The hydrophilic nature of the drug can hamper the achievement of high entrapment efficiency in nanosystems especially in lipid based nanocarriers. W/O microemulsion can provide promising alternative in which the hydrophilic drug is entrapped with the internal aqueous phase. The potential of w/o microemulsions of improving permeability and hence oral bioavailability of BCS class III have been investigated with promising results being published for drugs like acyclovir, metformin and fexofenadine [11–13]. The benefit of this strategy is augmented if the tested oil was natural with antidiabetic effect. The extract of nigella sativa seeds is claimed to exert antidiabetic activity [14–17]. Moreover, Nigella seeds are adopted as adjuvant to oral antidiabetic drugs [17, 18]. Accordingly, the objective of this study was to investigate nigella oil-based w/o microemulsion for oral delivery of linagliptin. The study was extended to fortify nigella oil with oils of known intestinal absorption enhancing ability. Isopropyl myristate (IPM) and capryol were selected for this purpose. Thus, phase diagrams were constructed for nigella oil alone or in combination with IPM or capryol. W/O microemulsion formulations were selected and investigated for enhanced oral delivery of linagliptin.

## Materials and methods

### Materials

Linagliptin was a gift sample from Amoun Pharmaceutical Industries Company, Elobour city, Egypt. Cellulose dialysis tubing (molecular weight cutoff 14,000 Da) and Streptozotocin were bought from Sigma Aldrich, MO, USA. Capryol PGMC (Propylene glycol caprylate) was gifted from Gattefosse, Lyon, France. Isopropyl myristate, Tween 60, potassium dihydrogen phosphate and sodium hydroxide were procured from Iso-chem, Cairo, Egypt. Hydrochloric acid and citric acid monohydrate were supplied by El Nasr Pharmaceuticals Chemicals Company, Cairo, Egypt. Tri sodium citrate was from Sigma for Pharmaceutical Industries, Quesna, Egypt. Acetonitrile (HPLC grade) was produced by Fisher Scientific (Loughborough, Lecis, UK). Nigella oil was purchased from Imtenan Company for Natural Products, Tanta, Egypt.

### Construction of pseudoternary phase diagrams

The oily phase was either nigella oil alone or nigella oil-permeation enhancer mixture. The enhancers employed were isopropyl myristate or capryol PGMC. These were separately mixed with nigella oil at weight ratio of 1:1. Tween 60 was applied as surfactant. The pseudo-ternary phase diagram was constructed in absence and presence of penetration enhancers. Water titration method was adopted at ambient temperature, 25 ± 1 ^o^C [19]. Briefly, nigella oil or nigella oil-permeation enhancer mixture was mixed with Tween 60 at increasing weight ratios (5:95, 10:90, 15:85, 20:80, 25:75, 30:70, 35:65, 40:60, 50:50, 60:40, 70:30, 80:20 and 90:10). Water was added gradually to each mixture under mixing. The titration involved continuous monitoring of phase change to determine the boundaries of the phase diagram. The phases were categorized as microemulsion, liquid crystal/gel or coarse emulsion. Single phase isotropic transparent liquids were selected as microemulsions. Opaque viscous mixtures showing birefringence with shining oil streaks were considered as liquid crystal/gel phases. Milky dispersions were seen as coarse emulsions [19].

### Preparation of microemulsions

W/O microemulsion systems were selected from the constructed phase diagrams. The selected microemulsion formulation included nigella oil or nigella oil-permeation enhancer blend, Tween 60 and water at weight ratio of 20:70:10, respectively (Table 1). The selected composition was away from the border of microemulsion to ensure stability of the system. Microemulsion was prepared by mixing the oily phase with surfactant followed by addition of water. Linagliptin was then added to the prepared microemulsion to provide drug concentration of 0.5 mg/ml.

**Table 1:** The composition and specifications of the tested w/o microemulsion formulations

Composition/ Characterization	F1	F2	F3
Nigella oil	20	10	10
Isopropyl myristate	----	10	----
Capryol PGMC	----	----	10
Tween 60	70	70	70
Distilled water	10	10	10
Droplet size from TEM (nm)	99 ± 15	83 *±* 29	47 *±* 8
Droplet size from zetasizer (nm) at zero time	104.9	121.2	86.4
Droplet size after storage at 4 ^o^C for 24 days	107.1	141.0	95.6
Droplet size after storage at 25 ^o^C for 24 days	103.6	143.7	89.5
Polydispersity index (PDI) at zero time	0.302	0.301	0.278
PDI after storage at 4 ^o^C for 24 days	0.382	0.375	0.377
PDI after storage at 25 ^o^C for 24 days	0.444	0.361	0.361
Viscosity (cp.)	612.5	517.7	379
Conductivity (µs/cm)^a^	7.15 *±* 0.13	7.55 *±* 0.23	6.43 *±* 0.14
Cumulative drug released (%)^b^	8.58 *±* 0.37	7.64 *±* 0.24	12.65 *±* 0.68

Linagliptin was added in the microemulsion at concentration of 0.5 mg/ml

^a^The electrical conductivity of sodium chloride solution in water was 2214.33 *±* 0.58 µs/cm

^b^The % Cumulative linagliptin released from aqueous dispersion was 69.14 *±* 1.12%

The values represent the mean *±* SD, *n* = 3. PGMC abbreviated for propylene glycol monocaprylate

### Transmission electron microscopy (TEM)

The morphological features and droplet size of microemulsion were determined by TEM (JEOL-JSM-1400 PLUS, Tokyo, Japan). Microemulsion was dropped on copper grid and a drop of ethanol was added. Staining employed uranyl acetate for 5 min followed by lead citrate for 2 min. The stained formulation was examined by TEM and selected fields were photomicrographed and droplet size was measured.

### Dynamic laser scattering spectroscopy (DLS)

Droplet size measurement employed DLS which also provides particle size distribution as polydispersity index (PDI). DLS was performed by a 90Plus Brookhaven zetasizer (Brookhaven Instrument Corp., Holsville, NY, USA) which works at fixed angle of 90^o^ and 25^o^C. Sample preparation involved dilution of the prepared microemulsions (1 in 100) using ethanol. The recorded results were in the form of average diameter (Z-average) and PDI.

### Measurement of viscosity

The flow behavior and viscosity of microemulsions were researched using HAAKE ViscoTester iQ (Thermo Fisher Scientific, Waltham, MA, USA).

### Conductimetric characterization of colloidal systems

The type of the tested microemulsion was determined by measuring the conductivity after replacing the water with 0.1% w/v aqueous sodium chloride. This salt was used to provide measurable readings for conductivity when present as external phase. Electrical conductivity determinations were achieved using Hanna-HI 2300 conductimeter.

### Physical stability study

The physical stability of selected microemulsions was assessed by monitoring the homogeneity of microemulsion after 30 min centrifugation at 4000 rpm (bench top centrifuge, Model 80–2, Taiwan). The same stress test was adopted for stability testing of microemulsion [20]. In addition, the microemulsions were stored at 4 and 25 ^o^C for 24 days at the end of which the formulations were subjected to visual inspection and droplet size measurement by DLS.

### In vitro drug release

Linagliptin release was tested through semipermeable membrane mounted between the receptor and donor compartments of vertical Franz diffusion cells [19]. The membrane was made of cellulose with MW cutoff 14,000 Daltons (cellulose tubing, Sigma Aldrich, St. Louis, MO, USA). Steady pore diameter was ensured by at least 24 h incubation in distilled water before mounting on the diffusion cells. The diffusion cells offer an average diffusional surface area of 2.27 cm^2^. HCl (0.1 N) was initially employed as receptor fluid for 3 h. After that, HCl was replaced by phosphate buffer pH 6.8. The tested formulation (2 ml) was loaded into the donor compartment and was occluded with aluminum foil. Sequential application of gastric and intestinal pH values in the receptor was previously adopted to monitor the release of linagliptin from lipid nanostructures [9]. Release was monitored in a thermostated water bath (37 ^o^C). Receptor fluid (5 ml in the acid phase and 8 ml in the intestinal phase) were periodically withdrawn and receptor was replenished after each sample to maintain constant volume. Linagliptin concentration in samples was quantified by HPLC after neutralization of the acidic samples with sodium hydroxide solution. The release profile is presented as the cumulative % released [21]. The release kinetics were computed by fitting the release data to zero order, first order and Higuchi kinetic models.

### High pressure liquid chromatography (HPLC)

Linagliptin concentrations were determined using HPLC (Agilent technologies 1260 infinity, DE, Germany). Quantification was at 226 nm using VWD 1260 UV detector. Potassium dihydrogen phosphate (20 mM, pH 3) was mixed with acetonitrile at 7:3 v/v and was pumped at flow rate of 1 ml/minute through a 4.6 × 150 mm Inertsil C18 HPLC column (GL sciences, Tokyo, Japan). Linagliptin stock solution was in ethanol which was suitably diluted with phosphate buffer (pH 6.8) to prepare standard concentrations. The sample (30 µl) was injected via autosampler (TCC 1260) for quantification. Data acquisition and treatment along with experimental conditions were through Agilent Open LAB Chem Station software.

### Investigation of the hypoglycemic effect of linagliptin

#### Induction of diabetes

The protocol of this investigation was initiated after approval from the ethical committee of Faculty of Pharmacy, Tanta University (approval number: TP/RE/8/23p-0044). This investigation was conducted on male albino rats weighing (250 *±* 20 g). Animal treatment and housing were based on the National Institute of Health guide for the care and manipulation of laboratory animals. The rats were fed on standard pellet diet with free access to drinking water. Induction of diabetes started by overnight fasting followed by single shot of freshly prepared streptozotocin (50 mg/kg body weight) in 0.1 M citrate buffer (pH4.5) given intra-peritoneal. Glucose solution (5% w/v) and food were allowed for the rats after injection to counteract potential hypoglycemia [22, 23]. Diabetes induction was assessed 48 h after administration of streptozotocin. This involved 2 h fasting and blood sample was taken from tail vein under ether anesthesia. Blood glucose level was determined by Gluco DR super sensor (Allmedicus, Gyeonggi, Korea). Diabetes was diagnosed in rats with fasting blood glucose level > 250 mg/dl.

#### Drug administration

Linagliptin (0.44 mg/kg) was orally administered as simple dispersion in water or after incorporation into microemulsion formulation.

#### Experimental design

The rats were divided into 9 groups (5 rats each). Group I (diabetic rats) received simple aqueous dispersion of linagliptin. Group II (diabetic rats) was treated with medicated microemulsion formulation containing nigella oil only as oily phase (F1), group III (diabetic rats) was treated with medicated microemulsion containing 1:1 blend of nigella oil and isopropyl myristate as oily phase (F2), group IV (diabetic rats) was treated with medicated microemulsion formulation containing 1:1 blend of nigella oil and capryol as oily phase (F3). Group V involved monitoring the blood glucose of non-diabetic rats maintained at the same experimental conditions of feeding and fasting. Group VI utilized diabetic rats receiving normal saline instead of formulations. Group VII used diabetic rats and received non-medicated F1. Group VIII used diabetic rats and received non-medicated F2. Group IX used diabetic rats and received non-medicated F3.

#### Procedures and sampling

On the day of experiments, diabetic rats were allowed free access to food and water for 30 min at the end of which food was banned and water was allowed throughout the rest of the experiment. Blood glucose level was determined two hours after food exclusion. This reading was considered zero-time reading and drug administration started immediately by feeding syringe. Blood glucose level was determined in blood samples collected 1, 2, 3, 4, 6, 8 and 24 h after drug administration. These levels were used to compute the amount of blood glucose reduction relative to zero-time blood glucose level. The results were graphically illustrated as a function of time. These plots were used to compute the area under blood glucose reduction curve (AUC). The time corresponding to maximum decrease in blood glucose level was taken as the (T_max_) and was computed from the rate of change in glucose level.

### Statistical analysis

The student`s t test was used to statistically evaluate the difference between groups.

## Results and discussion

### Pseudoternary phase diagram

**Fig. 1:**
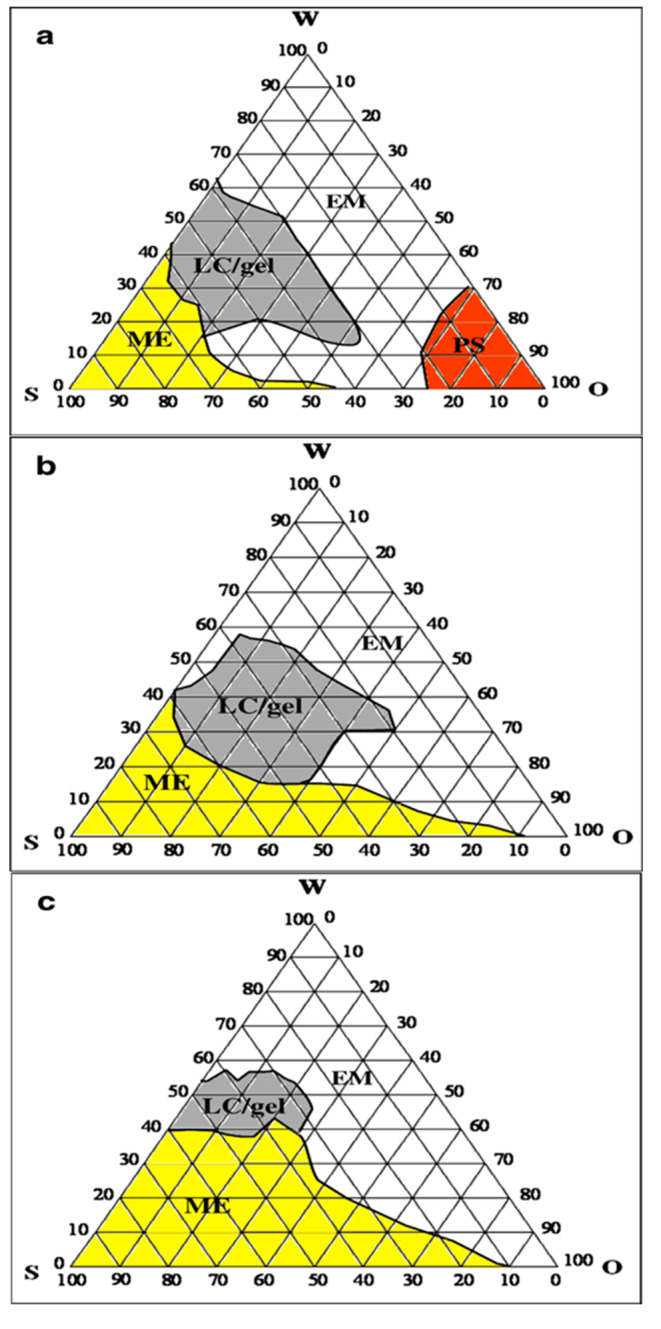
The pseudoternary phase diagrams of nigella oil (**a**), nigella oil/isopropyl myristate (**b**) and nigella oil/capryol (**c**) based systems with Tween 60 and water. W means water, O means oil or oil/penetration enhancer blends and S means surfactant. ME means microemulsion, LC means liquid crystal, EM means emulsion and PS means phase separation

Nigella oil was used as the principal oil in this study. Isopropyl myristate and capryol were selected as potential intestinal permeation enhancers. This selection was based on their miscibility with nigella oil. Tween 60 was selected as surfactant based on its ability to absorb the greatest amount of water when equally mixed with each oil system as the oil component of phase diagram. This strategy was previously utilized to select proper surfactant for construction of pseudoternary phase diagrams [19, 24]. The constructed phase diagrams are shown in Fig. 1. The phase behavior of nigella oil/Tween60 system differed with increasing water concentration. The recorded phases were microemulsion, liquid crystals (LC)/gel and coarse emulsion with phase separation being noticed at oil concentration approximately 70% w/w or higher. The LC/gel phases were collectively plotted in the phase diagram. The relative proportions of microemulsion, liquid crystal/gel and coarse emulsion zones were 13%, 21% and 57%, respectively of the total area of the phase diagram (Fig. 1). For nigella oil/IPM phase diagram, the same phases were seen but with different relative area. Importantly, no phase separation was recorded in the phase diagram. The area of this phase diagram included 26% as microemulsion zone with LC/gel covering 22%. The rest of the area was in the form of coarse emulsion. Likewise, the phase diagram of nigella oil/capryol mixture did not show any zone of phase separation. The microemulsion zone filled 41% with the LC/gel phase contracted to reach only 8% of the total area. The coarse emulsion zone was close to that seen in IPM/nigella system to record 51% of the phase diagram. The extension of microemulsion zone and contraction of LC/gel zone can be attributed to HLB of capryol (6, according to the supplier`s information). This HLB provides amphiphilicity for capryol which can result in fluid interface resulting in extended microemulsion zone. This amphiphilic nature allowed some researchers to employ capryol as cosurfactant [25]. Microemulsion formulations were selected from these phase diagrams. Selection involved regions away from the border of phase transition to guarantee stability of the system.

### Characterization of microemulsion formulations

**Fig. 2:**
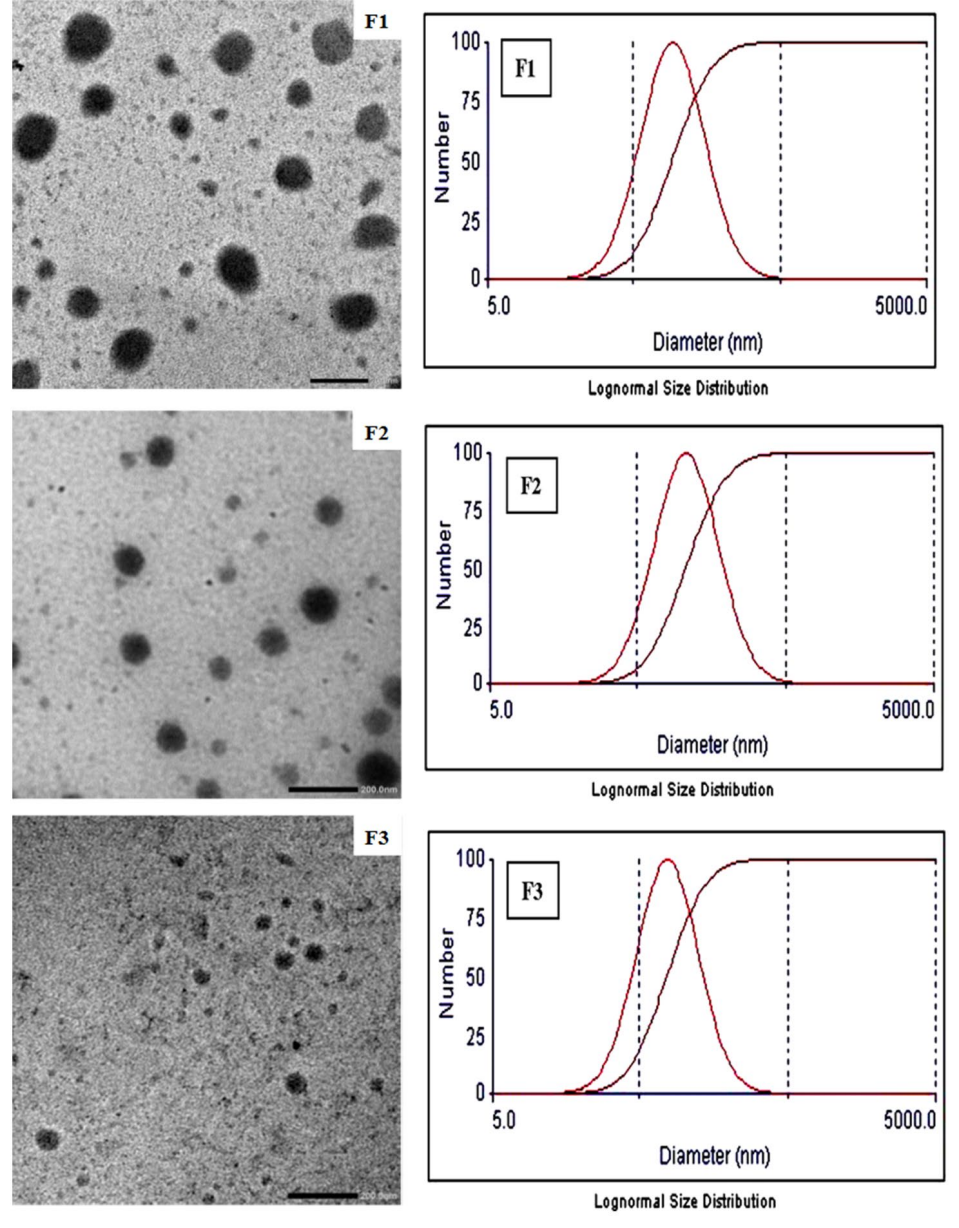
The transmission electron micrographs and zetasizer particle size distribution for different microemulsion formulations. Formulations details are presented in Table 1

The morphology and size of microemulsion droplets were researched by transmission electron microscopy. Figure 2 shows representative transmission electron micrographs of the tested formulations. The photomicrographs showed spherical microemulsion droplets with no aggregation with droplet size values within the nano-range. The average size values of microemulsion droplets (obtained from TEM) were 99 ± 15 nm, 83 *±* 29 nm and 47 *±* 8 nm for nigella, nigella/IPM and nigella/capryol microemulsions, respectively (Table 1). Similar morphological and size features were shown for microemulsion using TEM [26–28]. The droplet size was also determined by dynamic light scattering spectroscopy (DLS). Measurement of droplet size of net microemulsion formulations provided poor quality factor on the zetasizer and the samples required dilution. Dilution with water was excluded due to phase transition which is shown in the phase diagram and is documented in literature [29]. Ethanol was used as diluent due to its ability to widen the microemulsion zone by acting as cosurfactant [30]. The results of DLS showed droplet size values of 104.9, 121.2 and 86.4 nm for nigella, nigella/IPM and nigella/capryol microemulsions, respectively (Table 1). The variation in size from different techniques is expected considering the principle of size measurement in each strategy. For example, sample preparation in case of TEM involves staining and drying which can lead to possible shrinkage of the droplets, but DLS employs light scattering through the tested samples. Similar variation of size value was shown between TEM and DLS when researching particle size of nanosystems [31]. Interestingly, DLS reflected homogenous distribution of droplet size as noticed from the computed polydispersity indices which ranged from 0.278 to 0.302. In addition, graphical representation of particle size distribution indicated monodisperse diagram confirming the homogeneity which is expected for the thermodynamically stable microemulsion (Fig. 2).

**Fig. 3:**
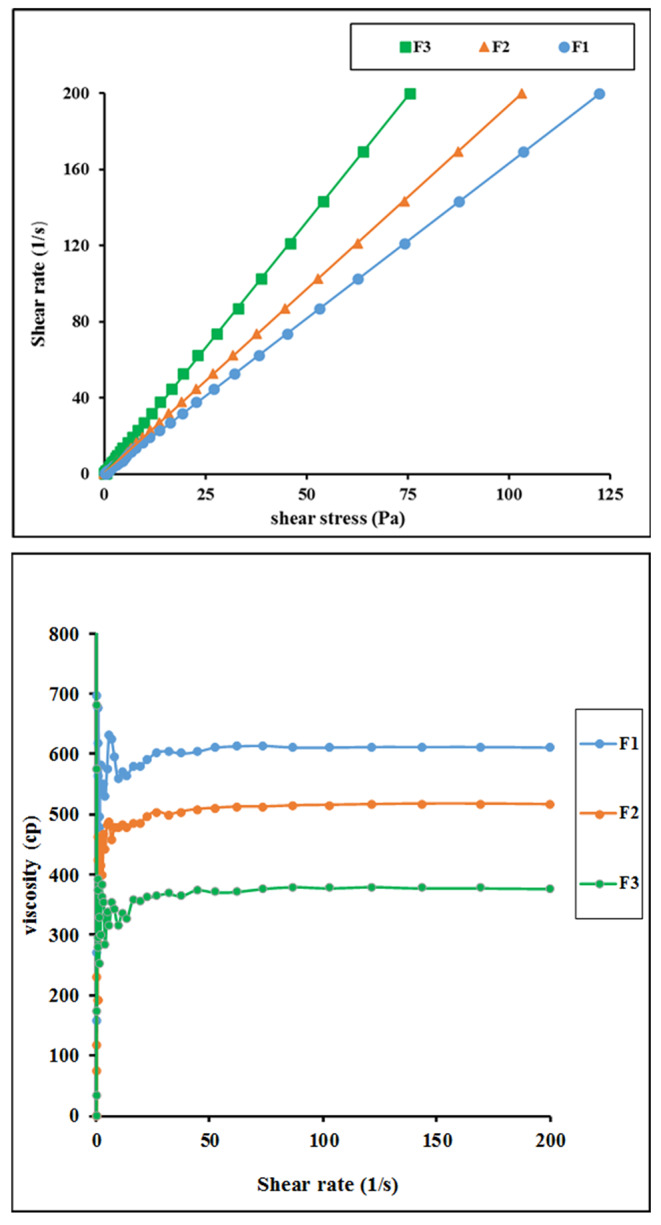
The Newtonian flow behavior of the tested microemulsion formulations. Formulations details are presented in Table 1

Figure 3 shows the flow behavior of the tested microemulsion formulations. All formulations exhibited Newtonian flow that is characterized by linear rheogram and constant viscosity values with increasing shear rate. The recorded viscosity depended on the composition of the formulation. Nigella-based microemulsion recorded a viscosity value of 612.5cp. For nigella/IPM microemulsion, the viscosity was reduced to reach 517.7cp with further reduction in viscosity being seen in case of nigella/capryol microemulsion which recorded a viscosity of 379cp (Table 1). The reduction in viscosity is parallel with the increase in the area of microemulsion zone in the phase diagram. This finding supports our explanation for extended microemulsion zone and reduced liquid crystalline zone in presence of IPM or capryol. Accordingly, fluidization of interfacial layer in presence of amphiphilic capryol can contribute to significant reduction in viscosity of microemulsion. Reduction in viscosity of microemulsion was reported after incorporation of cosurfactants [30].

The electrical conductivity values of different formulations prepared using 0.1% w/v sodium chloride solution as the aqueous phase were 7.15 *±* 0.13, 7.55 *±* 0.23 and 6.43 *±* 0.14 µs/cm for F1, F2 and F3, respectively. These values were lower than the electrical conductivity of sodium chloride solution which was recorded as 2214.33 *±* 0.58 µs/cm (Table 1). This reflects the location of aqueous phase in the internal compartment of microemulsion forming w/o microemulsion. This was expected taking into consideration the relative proportion of aqueous phase to oily phase. This confirmed the selection of formulation from w/o microemulsion.

Centrifugation assisted physical stability testing reflected the thermodynamic stability of the tested formulations which was indicated by absence of any change in the phase behavior of the tested systems even after 30 min centrifugation. The stability of the tested microemulsions was maintained even after storage at 4 and 25 ^o^C for 24 days. This was reflected from the physical appearance in addition to no significant change in the droplet size and polydispersity index of the formulations after storage (Table 1).

### In vitro drug release

**Fig. 4:**
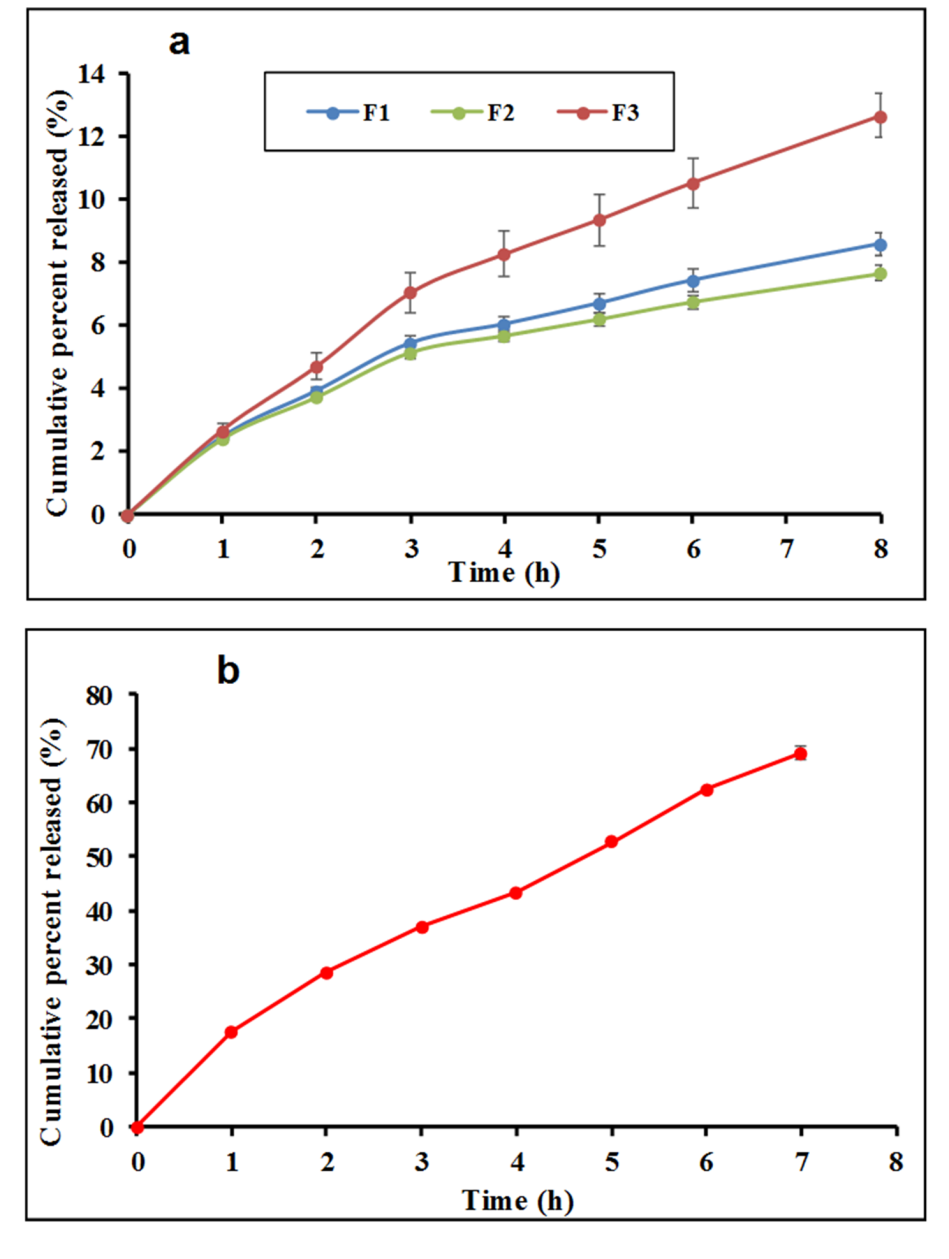
The release profiles of linagliptin from different microemulsion formulations (**a**) and from linagliptin dispersion (**b**). Formulations details are presented in Table 1

Figure 4 shows the release profiles of linagliptin aqueous dispersion and from microemulsion formulations containing 0.5 mg/ml of linagliptin. The release kinetics data are presented in Table 2. The simple aqueous dispersion underwent fast release of linagliptin with the release data fitting zero order release kinetics. This is expected taking into consideration the dissolution limited release of drug which diffuses through a membrane with fixed pore size. Liberation of drugs from aqueous dispersion/solutions was shown to follow zero order release kinetic [31, 32]. With respect to w/o microemulsions, the release data fitted better to Higuchi kinetics as indicated from the calculated values of the correlation coefficient (Table 2). Higuchi release kinetics suggest diffusion-controlled release which exists mainly in matrices and does not correlate with the fluid formulation. However, considering the localization of linagliptin in microemulsion which is expected to mainly occupy the internal aqueous phase, the drug can undergo diffusion from the internal to the external phase mimicking the matrix like structure. Another possible explanation for the recorded release kinetics may depend on possible back diffusion of the aqueous receptor into the microemulsion in the donor. This can result in thickening of the formulation to develop matrix like structure. Similar release kinetics were shown in fluid systems including multilamellar vesicular systems and w/o microemulsions [29, 33, 34]. Comparing the release behavior of different formulations, the release rate constant was considered. Based on this parameter, nigella oil/capryol-based microemulsion showed the fastest release rate compared with nigella oil or nigella oil/IPM-based systems (*P* < 0.05). Incorporation of IPM into nigella oil microemulsion did not alter the release rate significantly as indicated from the release rate constants (Table 2; *P* > 0.05). The difference in the release rate can be explained on the base of viscosity difference. This can justify the faster release rate in case of nigella oil/capryol formulation (F3) which was the least viscous (Table 1). However, the viscosity difference did not explain the absence of significant difference between F1 and F2 with respect to release rate. Accordingly, the phase behavior of each system was considered along with composition of each formulation which employed the same ratio of oil, surfactant and water. This consideration indicated that F1 and F2 are close to the gel zone suggesting easy thickening after mixing with the receptor fluid diffusing from the receptor medium. This justifies the recorded similar slower release rate from these formulations compared with F3 which is located away from the gel phase and requires larger amount of receptor to undergo significant thickening. Phase transition in the donor compartments was confirmed by visual inspection at the end of the release study. Similar justification is reported for the release data of drugs from microemulsions [29, 35].

**Table 2:** Correlation coefficient (R^2^) values and Higuchi release constant (K) values obtained from linear regression analysis of linagliptin release data fitted to different release kinetics models

	Zero order	First order	Higuchi	Higuchi release constant (K) (%/h^1/2^)
Linagliptin dispersion	0.9811	0.9444	0.9707	----
F1	0.9203	0.8507	0.9933	3.11 *±* 0.15
F2	0.8933	0.8296	0.9923	2.79 *±* 0.09
F3	0.9621	0.8542	0.9758	4.62 *±* 0.31

Formulations details are presented in Table 1

### In vivo evaluation of the hypoglycemic effect of the prepared microemulsion formulations

**Fig. 5:**
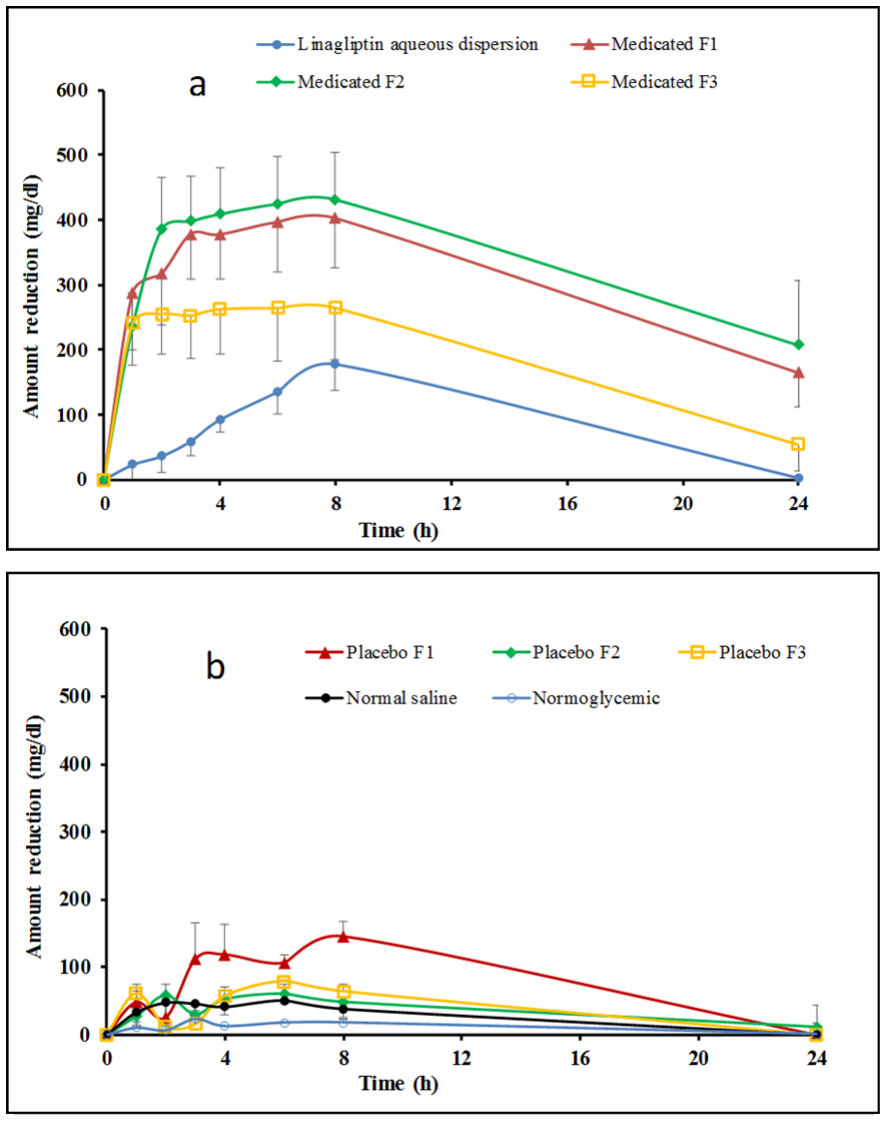
Cumulative amounts of blood glucose reduction as a function of time after oral administration of linagliptin aqueous dispersion or medicated w/o microemulsion formulations to diabetic rats (**a**) and after administration of saline or placebo (nonmedicated) w/o microemulsion formulations (**b**). Formulations details are in Table 1

Hypoglycemic response was considered as reliable indicator for monitoring in vivo efficacy of oral hypoglycemic agents. This parameter is considered acceptable for comparison between various formulations [23, 36, 37]. This strategy was employed after induction of diabetes in rats to monitor the hypoglycemic effect of linagliptin after oral administration as microemulsion or simple aqueous dispersion. For balanced experimental design, blood glucose levels of nondiabetic rats were monitored. In addition, blood glucose of diabetic rats receiving normal saline instead of formulations was monitored. Those receiving nonmedicated formulations were also assessed. The nondiabetic rats showed nearly steady blood glucose level throughout the experiments and resisted 10 h food restriction (Fig. 5; Table 3). Diabetic rats receiving normal saline showed lower ability to resist food restriction compared with normal rats. This was indicated from the significant increase in the area under blood glucose reduction curve (AUC), compared with that recorded in case of normal rats (Fig. 5; Table 3). Oral ingestion of linagliptin in the form of aqueous dispersion gradually reduced the blood glucose level of diabetic rats. The time at which the greatest reduction in blood glucose level (T_max_) was determined to be 4 h. This T_max_ value is in agreement with the results recorded by other investigators who monitored the pharmacodynamic and pharmacokinetics of linagliptin in diabetic rats [3]. The blood glucose level reduced at a slower rate after this T_max_ (Fig. 5). This gradual hypoglycemia can be accounted for the remaining plasma concentration of the drug in addition to the effect of food restriction. It is important to emphasize that the recorded effect of aqueous linagliptin dispersion was significantly higher (*P* < 0.05) than that noticed with diabetic rats receiving normal saline at the same experimental conditions. This confirms the hypoglycemic effect of linagliptin aqueous dispersion. Similar finding was shown in literature reports [3].

**Table 3:** The area under the blood glucose reduction curve (AUC) and T_max_ recorded for different groups

Group	Formulation	AUC (mg h/dl)	T_max_ (h)
Group I	Drug dispersion	2148.63 *±* 431.47	4
Group II	Medicated F1	7291.80 *±* 1555.72*	1
Group III	Medicated F2	8041.60 *±* 1861.30*	1
Group IV	Medicated F3	4481.90 *±* 1441.12	1
Group V	Untreated (normoglycemic)	204.67 *±* 21.06	---
Group VI	Normal saline	637.33 *±* 78.23	---
Group VII	Placebo F1	1457 *±* 155.27	3
Group VIII	Placebo F2	855.00 *±* 27.68	2
Group IX	Placebo F3	912.00 + 107.40	1

Values between brackets are S.E.M, *n* = 5. *Significantly different from drug dispersion (*P* < 0.05). AUC is area under blood glucose reduction curve. T_max_ is the time corresponding to maximum decrease in blood glucose level. Formulations details are presented in Table 1

The incorporation of linagliptin into the different microemulsions resulted in rapid and more enhanced anti diabetic effect after oral administration compared to linagliptin dispersion. This was reflected from the recorded T_max_ and AUC values recorded for groups treated with different microemulsions (Fig. 5; Table 3). For different microemulsion formulations, the maximum reducing effect was reached after 1 h. The rapid effect reflects rapid absorption from microemulsion formulations. Rapid absorption was indicated in case of all microemulsions irrespective to the composition of the formulation, but the extent of blood glucose reduction depended on this composition. The extent of reduction was assessed by monitoring the area under blood glucose reduction curve (AUC). Based on this, microemulsion formulations were ranked as nigella oil/IPM-based microemulsion (F2) > nigella oil-based microemulsion (F1) > nigella oil/capryol microemulsion (F3) > simple aqueous dispersion (Fig. 5; Table 3). Statistically speaking, the increase in the extent of blood glucose reduction was significantly higher after administration of F1 or F2, compared with simple aqueous dispersion (*P* < 0.05). F3 showed only a trend in increased reduction in blood glucose level compared with aqueous linagliptin dispersion (Table 3). To investigate the effect of formulation components on blood glucose levels, non-medicated formulations were administered to diabetic rats and the blood glucose was similarly monitored. Administration of non-medicated formulations showed some degree of reduction in blood glucose level with the extent of reduction arranged as nigella oil ME (F1) > nigella oil/capryol ME (F3) > nigella oil/IPM ME (F2). Interestingly, the effect of F1 which was contained the highest amount of nigella oil was significantly greater than normal saline. This suggests possible hypoglycemic effect for nigella oil and correlates with literature but the pronounced effect may need administration of larger amount of nigella oil [14–17]. The hypoglycemic effect of non-medicated systems was significantly lower than the corresponding medicated microemulsions suggesting that the recorded efficacy is mainly due to enhanced bioavailability of linagliptin with some contribution from nigella oil especially in F1.

The superiority of linagliptin containing microemulsion over linagliptin aqueous dispersion could be attributed to two main factors. Firstly, the possible benefits of nigella oil that is included as integral component of the microemulsions. These benefits include the potential anti diabetic effect of nigella oil that has been published by other investigators [14–17]. Furthermore, oleic acid which is a major constituent of nigella oil can enhance biological membrane permeability [17, 38, 39]. The second possible reason for the recorded superiority of microemulsion depends on the nanostructure of microemulsion which potentiates the effect of microemulsion components. The capabilities of w/o microemulsions to improve permeability and hence oral bioavailability of BCS class III drugs have been investigated with promising results being published for drugs like acyclovir, metformin and fexofenadine [11–13]. The investigators attributed the beneficial effects of microemulsions to the combined effects of membrane fluidizing power of microemulsion components and the potential of nanosized droplets to intercalate between the microvilli providing high local concentration. This combined effect can subsequently hasten drug absorption [11, 19, 40–42]. Translymphatic absorption of drug loaded microemulsion is another possibility [13, 19, 41, 43]. Taking the above explanation into consideration, the superiority of IPM/nigella oil containing microemulsion (F2) can be linked with the extensive membrane fluidizing power of IPM compared with other microemulsion components [44]. The features of IPM together with the positive effects of nigella oil and nanoarchitecture of microemulsion provides summative explanation for the superiority of IPM/nigella-based system over capryol/nigella-based formulation. Despite of the reported permeation enhancing activity of capryol [45], the recorded results suggest that capryol has weaker enhancing effect than IPM or nigella component. However, this supposition requires future verification. Overall, the nanostructure of w/o microemulsion seems to be the key factor in enhanced oral availability of linagliptin with the composition providing additional effect which requires further investigations to test wider range of composition variables. It is important to emphasize that the tested formulation contained up to 70% Tween which raises question marks about its toxicity. Formulations containing up to 81% Tween are reported in literature [46]. The toxicity of microemulsion formulations containing different surfactants was investigated and formulations containing Tween 80 was shown to provide the least gastrointestinal mucosal damage [47]. Based on these reports, the use of the current formulation can be tentatively justified and future verification is required.

## Conclusion

Nigella oil was successfully developed as microemulsion in absence and presence of isopropyl myristate or capryol. The prepared formulations were in nanosized range with Newtonian flow behavior. Incorporation of linagliptin in w/o microemulsion controlled the release rate to be slower than simple aqueous dispersion. Nigella oil/capryol-based microemulsion showed faster release compared with nigella oil or nigella oil/IPM-based systems. Incorporation of linagliptin in nigella oil based microemulsions resulted in fast and more intense reduction in blood glucose level of diabetic rats compared with aqueous linagliptin dispersion. Presence of capryol in ME did not add significantly to the effect of nigella oil-based ME. The maximum effect was recorded for nigella oil/IPM-based system. The study introduced w/o microemulsion as promising carrier for enhanced oral absorption of hydrophilic drugs like linagliptin.

## Data Availability

Raw data will be available upon request.
